# Evaluation of a primary care-based collaborative care model (PARTNERS2) for people with diagnoses of schizophrenia, bipolar, or other psychoses: study protocol for a cluster randomised controlled trial

**DOI:** 10.3399/BJGPO.2021.0033

**Published:** 2021-05-19

**Authors:** Humera Plappert, Charley Hobson-Merrett, Bliss Gibbons, Elina Baker, Sheridan Bevan, Michael Clark, Siobhan Creanor, Linda Davies, Rebecca Denyer, Julia Frost, Linda Gask, John Gibson, Laura Gill, Ruth Gwernan-Jones, Pollyanna Hardy, Joanne Hosking, Peter Huxley, Alison Jeffrey, Benjamin Jones, Steven Marwaha, Vanessa Pinold, Claire Planner, Tim Rawcliffe, Siobhan Reilly, Debra Richards, Lynsey Williams, Max Birchwood, Richard Byng

**Affiliations:** 1 School of Psychology, University of Birmingham, Birmingham, UK; 2 Community and Primary Care Research Group- Faculty of Medicine, University of Plymouth, Plymouth, UK; 3 Coventry and Warwickshire Partnership NHS Trust, Coventry, UK; 4 Institute of Health Research, University of Exeter, Exeter, UK; 5 Birmingham Clinical Trials Unit, Birmingham University, Birmingham, UK; 6 London School of Economics and Political Science, London, UK; 7 Exeter Clinical Trials Unit, University of Exeter, Exeter, UK; 8 Peninsula Clinical Trials Unit, University of Plymouth, Plymouth, UK; 9 Division of Population Health - Health Services Research & Primary Care, University of Manchester, Manchester, UK; 10 Psychiatry, University of Manchester, Manchester, UK; 11 McPin Foundation, London, UK; 12 School of Health Sciences, Bangor University, UK; 13 Lancashire Care NHS Foundation Trust, Preston, UK; 14 Faculty of Health Studies, University of Bradford, Bradford, UK; 15 Warwick Medical School, University of Warwick, Warwick, UK

**Keywords:** Randomized Controlled Trial, Protocol, Collaborative Care, Schizophrenia, Bipolar Disorder, Psychotic Disorders

## Abstract

**Background:**

Current NHS policy encourages an integrated approach to provision of mental and physical care for individuals with long term mental health problems. The ‘PARTNERS2’ complex intervention is designed to support individuals with psychosis in a primary care setting.

**Aim:**

The trial will evaluate the clinical and cost-effectiveness of the PARTNERS2 intervention.

**Design & setting:**

This is a cluster randomised controlled superiority trial comparing collaborative care (PARTNERS2) with usual care, with an internal pilot to assess feasibility. The setting will be primary care within four trial recruitment areas: Birmingham & Solihull, Cornwall, Plymouth, and Somerset. GP practices are randomised 1:1 to either (a) the PARTNERS2 intervention plus modified standard care (‘intervention’); or (b) standard care only (‘control’).

**Method:**

PARTNERS2 is a flexible, general practice-based, person-centred, coaching-based intervention aimed at addressing mental health, physical health, and social care needs. Two hundred eligible individuals from 39 GP practices are taking part. They were recruited through identification from secondary and primary care databases. The primary hypothesis is quality of life (QOL). Secondary outcomes include: mental wellbeing, time use, recovery, and process of physical care. A process evaluation will assess fidelity of intervention delivery, test hypothesised mechanisms of action, and look for unintended consequences. An economic evaluation will estimate its cost-effectiveness. Intervention delivery and follow-up have been modified during the COVID-19 pandemic.

**Conclusion:**

The overarching aim is to establish the clinical and cost-effectiveness of the model for adults with a diagnosis of schizophrenia, bipolar, or other types of psychoses.

## How this fits in

The current NHS Community Mental Health Transformation policy (NHS England 2019) aims to ensure all those with psychosis and a need for care are supported, and for much of this to be carried out as a collaboration between primary care and third sector organisations. Previously, the policy focus was specialist mental health care via community teams and then discharge to primary care for those with less, but often still significant, need, thus presenting a significant divide between primary and secondary care. The authors have completed 2.5 years of preparatory work to understand the nature, strengths, and limitations of the current status of primary–secondary care collaboration and, alongside service users, have developed and piloted a primary care-based collaborative care model. PARTNERS2 is the first trial of that model to be carried out in the UK.

## Introduction

This article presents the protocol (version 7.3, 18.08.2020) for the PARTNERS2 (develoPing intergrAted primaRy care for paTieNts with sERiouS mental illness) cluster randomised controlled trial (RCT) to assess the clinical and cost-effectiveness of a primary care-based collaborative care model for people with diagnoses of schizophrenia, bipolar, or other psychoses. Such individuals are recognised as having considerable unmet physical, emotional, and social needs.^1^ The current NHS Community Mental Health Transformation policy (NHS England 2019) aimed to ensure all those with psychosis and a need for care are supported, and for much of this to be carried out as a collaboration between primary care and third sector organisations. Previously, the policy focus was specialist mental health care via community teams and then discharge to primary care for those with less, but often still significant, need, thus presenting a significant divide between primary and secondary care.

PARTNERS1, which ran during this previous policy, found that nearly a third of people in the UK with a diagnosis of schizophrenia, bipolar, or other psychoses were seen only in primary care.^1^ Poor continuity of care and lack of information exchange between primary and secondary care created barriers to effective support. To overcome these challenges, the PARTNERS model was developed to improve collaboration between secondary care, primary care, and the service user.

### Feasibility and piloting in the PARTNERS2 programme

Initial phases of PARTNERS2 focused on understanding current care pathways, developing the intervention, and addressing trial science questions. Different phases and timescales of the overall programme are summarised in Table 1. This protocol was written during the delivery and follow-up phase of the trial; use of past and present tense in the paper indicate activities at the time of writing.

**Table 1.** Programme and trial phases and timelines

**Stage and key aims**	**Duration**	**Key dates**
**Develop** **f** **easibility stage**Aims:To develop and refine the intervention modelTo agree outcome measuresTo test recruitment processes	24 months	2014–2016
**Randomised controlled trial** **: internal pilot**Aims:To further test feasibility of both recruitment and the intervention delivery for patients and GP practices.To assess recruitment against objectives: GP practice recruitment rates – 8 GP practices per site (24 total) Participant eligibility rates – 24 participants per site (72 total) To assess delivery and safety of intervention—for example, intervention delivery (care partners in place) and adverse events such as crisis care (home treatment teams), and admissions (psychiatric).To review initial sample size target of 336 participants	6 months	Trial started: 1/10/2017Trial registered: 16/10/2017First participant recruited: 8/6/2018
**R** **CT** **(completion of RCT following internal pilot):**Aim:To establish the clinical and cost-effectiveness of primary care based collaborative care for people with a clinical diagnosis of schizophrenia, bipolar, or other types of psychosis^a^	Completion of recruitmentFollow up: 10–12 months	Recruitment end date: 28/2/2020Follow up end: 28/12/2020Data collection end date:31/3/2020Study end date: 30/04/2021

a Revised recruitment target of 204 participants with a diagnosis of schizophrenia, bipolar, or other types of psychosis from ~34 clusters (GP practices), based on prespecified sample size recalculation (December 2019) and funder requirements (January 2020).

RCT Randomised controlled trial

A multi-site notes review was carried out to understand the relationship between primary and secondary care. The intervention was co-produced by clinical academics, researchers, and people with lived experience of ongoing mental health needs. The latter group were engaged through three Lived Experience Advisory Panels (LEAPs) advising the programme. The intervention was founded on Wagner’s Chronic Care Model,^2^ principles of personal recovery,^3^ and coaching for mental health.^4^ The programme theory and practice were iteratively developed utilising a realist approach,^5,6^ wider literature, primary data, and stakeholder input.^7^ The intervention was ‘fixed’ after the refinement in this feasibility stage.^8^

Approaches to recruitment were tested iteratively involving flexibility for non-responders and support from NHS employed researchers for pre-consent work (initial identification and approach by letter and telephone) in primary care. A core outcome set for use in bipolar disorder trials was developed.^9^ To account for the wider psychosis target population in PARTNERS2 and the nature of the intervention, an additional stakeholder consultation was undertaken to select outcomes and measures. QoL was selected as the most important outcome domain. Relevant measures of QoL were reviewed and the Manchester Short Assessment Quality of Life (MANSA) was selected on the basis of being clinically relevant to the target population and as potentially amenable to change by the intervention. The MANSA has good validity and reasonable internal consistency.^10^

### Challenges during trial set up, internal pilot, and COVID-19

Health Research Authority guidance changed while planning the trial, which meant that the flexible approaches developed during the conduct of the pilot phase were initially not permitted due to confidentiality concerns. Some components (for example, practices supported in pre-consent identification and approaches by NHS-employed researchers) were reinstated following Health Research Authority amendments once recruitment was shown to be slow. Changing circumstances in host NHS Trusts and delays in trial set-up led to withdrawals from the study of one trust during set-up, and the Lancashire Care Foundation Trust during the early phase of recruitment, resulting in the need to identify new host trusts. Although not all recruitment targets had been met, recruitment in two trusts (Cornwall and Plymouth) had been shown to be successful, and thus the trial was continued pending a delayed sample size review carried out in late 2019. As detailed in Table 1, this review led, in December 2019, to the recommendation from the trial oversight groups (Data Monitoring Committee [DMC] and Trial Steering Committee [TSC]) for a reduction of the original sample size. In January 2020, the funder requested recruitment to stop in order to complete the trial within the funding envelope and with a change in power (see sample size section below).

Soon after recruitment had closed, and partway through intervention delivery and follow-up data collection, the COVID-19 pandemic and national lockdown in the UK (March 2020) had a sudden impact on the trial. In consultation with the TSC and funder, the research team adapted methods for intervention delivery, follow-up data collection, and the ongoing process evaluation. These adjustments are outlined in Table 2. The adapted methods were tested for an 8-week period. In light of the success of this adaption, the continued uncertainty surrounding the pandemic, and risk assessments for both service users and practitioners, the decision was made to continue remote delivery, where possible, of both the intervention and follow-up, and process evaluation data collection until the end of the trial. Face-to-face delivery and data collection were used when safe and preferred by participants.

**Table 2.** Adjustments to the trial due to COVID-19 restrictions (8-week trial phase)

**Challenges**	**Solutions**
Adapting the intervention to remote delivery	Delivery via telephone or video conferencing software. Intervention practitioners received training on using video conferencing software and delivering interventions remotely. Coaching and goal-setting were adjusted to be appropriate to a lockdown environment. The majority of participants found it acceptable to continue the intervention after adaption to remote delivery. Practitioners delivering the intervention reported they were able to continue collaborating with primary care by remote means.
Collecting data remotely	Follow-up data and process evaluation data collected via telephone, video conferencing, or post. For the secondary outcome of time use, data collection was adjusted to include activities participants conducted remotely, such as attending church via video conferencing.
Understanding the feasibility and acceptability of continuing the intervention and data collection remotely. This included acceptability to participants and the feasibility of collaborating with primary care.	8-week trial phase, including rapid realist evaluation. This realist evaluation considered: the experiences of the intervention practitioners delivering the intervention during COVID-19 restrictions; and during routine audio-assisted recall interviews with service users, exploring their experiences of engaging with the intervention by remote methods.

## Method

### Objectives

The PARTNERS2 cluster RCT tests a collaborative care model for people diagnosed with bipolar, schizophrenia, and other forms of psychosis in addition to modified usual care, in comparison with usual care alone. The trial aims to understand the benefits of refocusing mental healthcare for the target group into primary care through provision of a coaching-based collaborative model to assist individuals with their recovery. The primary hypothesis is intervention arm participants will have improved QOL compared those receiving usual care alone.

The secondary hypotheses are that, in comparison with usual care only participants, intervention arm participants will have improved:

1. hours per week spent in structured activity;
2. mental health wellbeing;
3. physical healthcare;
4. increased experiences of personal recovery.

An economic evaluation will assess the costs, outcomes, and relative cost-effectiveness of the intervention (see Health economics evaluation section below). The study includes a comprehensive mixed-methods process evaluation to assess fidelity to the model, test the programme theory, and gain a more in-depth understanding of the intervention in practice in relation to implementation barriers and facilitators.

### Patient and public involvement

Several different patient and public involvement approaches are embedded into the trial design. The independent steering group and the DMC have patient and carer members. The study sites all recruited local LEAPs and service user posts were created, coordinated by The McPin Foundation. This means that many aspects of the trial have been shaped by expertise from experience, as follows:

- The original research design and research question was co-designed with patients, including a co-applicant.
- The intervention and guidance manuals have all been developed and reviewed with the three LEAPs. Care partner training was co-designed by the LEAPs. Service user researchers delivered elements of this training.
- The trial outcome measures were selected through a sub-study, which also developed a core outcome set for bipolar. This involved input from the three LEAPs. Patients and carers were part of the final decisionmaking workshop where trial outcomes were agreed.
- The LEAPs met every 3 months. They reviewed all recruitment materials and contributed to the development of interview schedules; produced a study specific website with video content to aid participant understanding of what taking part would involve; and were kept up-to-date throughout via meetings and newsletters. Service user researchers have been involved in recruitment of participants and undertaking other study roles alongside traditionally recruited research assistants. The role of the LEAPs and service user researchers in the PARTNERS2 programme is the subject of a separate article.^11^
- Dissemination will include input from the LEAPs. A LEAP-led dissemination event and accessible dissemination materials for participants, including a video and infographic, are planned.

### Trial design and setting

This is a cluster randomised controlled superiority trial, with randomisation at GP practice level. All participants continue to receive their usual care. The trial is registered as ISRCTN 95702682. See Table 1 for registration details.

The trial was conducted in GP practice research sites within four English geographical regions: Birmingham and Solihull, Cornwall, Plymouth, and Somerset. This represents a varied cross-section of society, including urbanity/rurality, and different levels of ethnic diversity.

GP practices were assessed for capacity and capability to deliver the trial. This included willingness to physically host the Care Partner, including computer access (key intervention role, see intervention description below), and support data collection requirements.

### Study population and recruitment of participants

**Figure 1.**
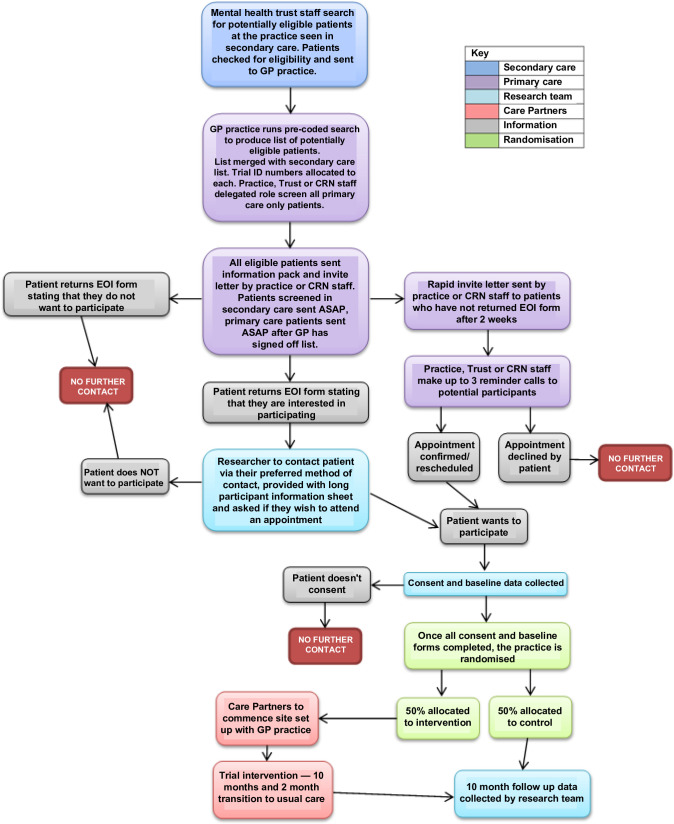
Flowchart detailing recruitment of research participants process. ASAP = as soon as possible. CRN = clinical research network. EOI = expression of interest.

The study recruited adults with a diagnosis of schizophrenia, bipolar, or other psychoses who either: are seen in secondary care and considered to of be lower risk/need; or are seen only in primary care (further details in Table 3). Figure 1 provides detail of the recruitment process, outlined below.

**Table 3.** Trial entry criteria for research participants

**Inclusion** **c** **riteria** Registered with a participating GP practice (with at least six individuals coded as having severe mental illness), which refer into recruited community mental health teams, within one of the four region NHS Trusts: Birmingham and Solihull Mental Health Foundation Trust, Livewell South West (Plymouth), Cornwall Partnership NHS Foundation Trust, or Somerset Partnership NHS Foundation Trust. Aged ≥18 years A clinical diagnosis of schizophrenia, bipolar, or other types of psychoses Evidence for care need in relation to this diagnosis in previous 2 years (automatic for those in secondary care; assessed from notes for primary care only). **Exclusion** **c** **riteria** Inability to understand English (or access translation services) Inability to give informed consent Significant need requiring ongoing secondary multi-disciplinary care (such as those meeting criteria for assertive outreach or early intervention functions, and therefore defined as higher, rather than lower, risk or need) Currently receiving home crisis care or care in an inpatient or secure setting Those excluded at the discretion of GPs, if it is felt that inclusion in this trial is not within the best interests of their patient Currently participating in a cognitive behavioural therapy, or a psychosocial or medicinal trial for psychosis or bipolar Individuals with a primary diagnosis of dementia receiving secondary care for dementia Individuals with a primary diagnosis of learning disability receiving care from secondary care for learning disability Individuals with ongoing significant and chaotic substance or alcohol misuse, making engagement with trial and intervention problematic

CRN Clinical Research Network EOI Expression of Interest ASAP As soon as possible

Potential participants seen in secondary care were identified from electronic records. Individuals were checked for eligibility by experienced secondary care clinicians and suitability confirmed by primary care staff. Potential participants seen only in primary care were identified from searches of electronic health records that were then checked for eligibility by primary care or clinical research network staff.

After eligibility checks, information packs were posted to potential participants who were asked to return expressions of interest (or wish to decline). Interested participants were contacted by their preferred method. Arrangements to meet at the practice or home were made. They could give informed consent either at the initial meeting or at a later point; participants could choose to complete baseline measures in multiple meetings.

Non-responders were sent an appointment letter, inviting them to an appointment with a researcher. Where capacity allowed, practice or secondary care staff telephoned the participant to remind them (maximum of three attempts). The appointment allowed researchers to discuss the study face-to-face; if, at the end of the discussion, the potential participant wished to consent to participate in the study this could take place a) in the same meeting, if there was time, or b) at an additional meeting if there was not.

Participants were provided with a £10 high street shopping voucher and thank you card as a gesture of gratitude on completion of each data collection appointment (a total of £20 for baseline and follow-up appointments) and for any qualitative interviews. All recruitment documentation was designed in consultation with LEAPs.

### Randomisation and allocation concealment

Practices were randomised to control and intervention groups on a 1:1 basis, stratified by recruiting region and estimated practice psychosis population (small and large), determined by the number of adults categorised as MH001 according to the Quality and Outcomes Framework register 2017/18 (median 70 patients).^12^ Practices were classified as ‘small’ if the MH001 list was <70 and ‘large’ if ≥70 adults.

The Clinical Trials Units (CTU) conducted the randomisation procedure. Allocation was concealed from participants, researchers, and practices until recruitment was complete within each practice (cluster). Researchers then requested unmasking.

After unmasking of a practice, the practice and its participants were informed of the allocation by letter, or the participant’s preferred method of contact. For logistical reasons, researchers conducting follow-up data collection could not be masked. Those conducting statistical analyses are masked from assignment.

### Intervention

The PARTNERS service places a trained secondary mental healthcare worker in primary care (a ‘Care Partner’). Care Partners work collaboratively with the participant, primary care, secondary care, and other organisations, aiming to improve the participant’s QOL, mental health, and physical health care. Place and method of contact was flexible, with contact once every 2 weeks at the start, stepping down to once every 3 months for some, but stepping up to referral into secondary care if required. The manualised model (manual available from authors on request) of person-centred care aims to improve the emotional, social, and mental and physical health needs of people with severe mental illness (SMI). The Care Partners work with participants to develop a shared understanding and utilise coaching techniques with the intention of encouraging participants to:

- Be more confident and proactive about their health through supporting participants to improve their self-management skills.
- Achieve personal goals related to their health or other aspects of their lives on their recovery journey.

Participants receive the intervention for up to 12 months, including a 2-month transition period back to usual care only. Participants on the secondary care caseload remain so for the duration of the trial. For those receiving the intervention, the intensity of usual care was reduced according to a protocol specific to each trust, to the extent that was possible based on their individual risk, governance, and Care Programme Approach procedures.

Care Partners are mental health workers with a variety of backgrounds, and include occupational therapists, social workers, psychiatric nurses, and support workers. They received 2–3 days initial training in the PARTNERS2 collaborative care model, top-up training throughout the study from PARTNER2 study team members, and supervision from trained supervisors. Supervisors included community mental health team managers, mental health nurses, and psychiatrists who all received training in the PARTNERS2 model. GP co-chief investigator (RB) provided cover for supervision during periods of illness or absence.

### Control group

All participants allocated to the control arm of the trial continue to receive usual care, either within primary care only or also with secondary care. This comparator reflects the unmet treatment need that the intervention aims to resolve. Such care is proactive in secondary care, but with variable practitioners and frequency. Primary care includes an annual review and otherwise tends to be reactive, including mental and physical care as clinically indicated.

### Outcome measures

The trial primary outcome is QOL at the primary endpoint, as measured by the MANSA. This is a self-complete questionnaire comprising both objective and subjective questions across different life domains (work and education, personal finances, leisure activities, social life, living situation, family life, personal safety, and health). Objective items (for example, employment status) are described separately and not aggregated. Satisfaction is rated on a 7-point Likert scale (1 = negative extreme, 7 = positive extreme) and scorings are processed to provide three different types of results: separate domain ratings; an overall MANSA score, assessing QOL (the primary outcome); and a general (single item) QOL rating. Staff undertaking data collection were trained in using the measure. The MANSA was collected at baseline and follow-up.

Secondary outcome measures were assessed at baseline and follow-up (listed in Table 4). Data collection forms are available from the authors on request.

**Table 4.** Schedule of assessment for practices and participants

**Activity**	**Pre-baseline**	**Baseline**	**Pilot phase** **6** months	**Month 10** **±30** **days**
Eligibility check	*x*			
Valid informed consent	*x*	*x*		*x*
**Data to inform if full-scale RCT is feasible**				
Practice recruitment rates	*x*	*x*	*x*	*x*
Practice withdrawal rates			*x*	*x*
Participant eligibility rates	*x*	*x*	*x*	*x*
Patient recruitment rates		*x*	*x*	
Participant withdrawal rates			*x*	*x*
**Outcome measures**				
Quality of life (MANSA)		*x*		*x*
Time use (ONS Time Use Survey)		*x*		*x*
Personal recovery: questionnaire about the process of recovery (QPR-15)		*x*		*x*
General health status and quality adjusted life years: EuroQol (EQ-5D-5L)		*x*		*x*
Mental wellbeing: Warwick–Edinburgh mental wellbeing scale		*x*		*x*
Capability measure and quality adjusted life years: ICEpop capability measure		*x*		*x*
NHS resource use: records audit and economic interview				*x*
Experience of care: brief INSPIRE		*x*		*x*
Healthcare monitoring				*x*
**Safety variables**				
Admissions (psychiatric)				*x*
Crisis care (home treatment)				*x*
**Process evaluation**		*x*		*x*
**Impact of COVID-19**				*x*

MANSA = Manchester short assessment quality of life. ONS = Office of National Statistics. QPR = Questionnaire for Personal Recovery. RCT = radomised controlled trial.

NHS and social care service use for the preceding 3 months prior to baseline and follow-up was assessed to estimate service cost. The period of 3 months was chosen to optimise accuracy of recall and scope of service use. Data includes:

- outpatient contacts;
- inpatient stays;
- primary care consultations; and
- other health and social care contacts;

The following secondary outcomes were assessed at 10 months only from primary care and secondary care records:

- Healthcare monitoring to assess which of the following have been received by the patient within their GP practice:
  - annual health check;
  - blood pressure, lipids, glucose assessed, and whether treatment was changed; and
  - lifestyle interventions (for example, smoking cessation, alcohol, and dietary guidance)
- Safety variables:
  - number of crisis care episodes and days under home treatment; and
  - mental health admissions and number of days’ inpatient;

#### COVID-19 sub-study

Participants who experienced COVID-19 restrictions during their involvement in the study in both arms are asked additional questions at follow-up. This capitalises on the opportunity to understand how lockdown has impacted on the participants’ mental health, access to physical health care, and the impact of social distancing on usual activities.

#### Retention

Researcher rapport, flexibility in contact, and thank you vouchers were used to enhance retention. Where participants withdraw from either the intervention or the study, reasons for withdrawal will be captured via quantitative and qualitative data where possible.

### Sample size

The initial sample size for the full trial was based on detecting a mean between-group difference of 0.45 points in the overall MANSA score. Assuming a standard deviation (SD) of 0.90, this is equivalent to detecting half a SD. This standardised effect size corresponds to a medium to large effect size, and in line with the target difference for the DIALOG+ trial.^13^

To detect this target difference, and assuming SD of 0.90, coefficient of variation of cluster size of 0.74 and intra-cluster correlation of 0.05, the recruitment target for the full trial was originally 336 participants across around 56 practice clusters. This assumes an mean average of 6 participants per cluster, 20% drop out at the individual participant level, and 10% drop out at the cluster level.

The trial protocol included an interim blinded review of the assumptions underpinning the original sample size calculation. The pre-specified review was based on data from 39 participants with complete baseline and follow-up primary outcome data, and explored the a priori assumption of a correlation of 0.5 between baseline and follow-up MANSA scores, but which had not been included in the original sample size calculation. From the blinded review, the point estimate of this correlation was 0.69 (80% confidence interval [CI] = 0.56 to 0.79), and, as such, it was deemed appropriate to conservatively allow for a correlation of 0.5 in a revised sample size calculation. Retaining all the other original assumptions of the sample size calculation indicated a recruitment target of 270 participants from around 45 GP practices, to achieve 90% power or 204 participants from around 34 GP practices to achieve 80% power, to detect the pre-specified between-group difference of 0.45 units. In December 2019, both the DMC and TSC approved a revised recruitment target of 270 participants.

In January 2020, the study funder mandated that trial recruitment be terminated at the end of February 2020, in line with the recruitment timescales for the funded programme and based on evidence that recruitment numbers would likely provide approximately 80% power (achieving 270 participants would have required a significant costed extension). Following the funder’s decision, the aim was to recruit 204 participants, from approximately 34 GP practices, to achieve 80% power to detect the pre-specified target difference of 0.45 units, based on the underpinning assumptions of SD of 0.9, mean cluster size of 6 participants, coefficient of variation of 0.74, intra-cluster correlation of 0.05, correlation between baseline and follow-up MANSA scores of 0.5, and allowing for 20% drop out at the individual participant level and 10% drop out at the cluster level following randomisation.

### Serious adverse events

The risk of harm associated with trial procedures and the intervention are considered to be low, and therefore only serious adverse events (SAEs) are being collected. Investigators collate and report all SAEs that meet the following definition:

Any untoward medical occurrence or effect that:

- results in death;
- is life threatening;
- requires hospitalisation or prolongation of existing hospitalisation;
- results in persistent or significant disability or incapacity;
- is a congenital abnormality or birth defect; or
- is otherwise considered medically significant by the investigator. This will include:
  - crisis care (contact with Home Treatment Team), and
  - self-harm.

Planned hospital admissions or attendances for less than 24 hours are not reported. SAEs are recorded in the source data and on the case report form, and are reported to the trials office within 24 hours of awareness of the event by completing and submitting an SAE form.

### Data management and statistical analysis

Processes to ensure the accuracy of the data are detailed in the trial data management plan. Coding and validation have been agreed between the trial team, statistician, and the trial programmer.

Data from case report forms are entered onto the database by researchers and/or CTU staff in accordance with working instructions, and data queries are managed through the use of data clarification forms. Corrections which are self-evident will be agreed with the Chief Investigator (CI).

Statistical analyses are pre-specified and a detailed statistical analysis plan will be agreed by the DMC and signed off by the independent TSC statistician prior to locking of the database. This plan will be available from the authors on request. Results will be reported in line with relevant Consolidated Standards of Reporting Trials (CONSORT) guidelines.^14–16^ The primary analyses will be undertaken according to a modified intention-to-treat principle, including all participants who are randomised and who provide valid outcome data at baseline and 10-month follow-up. For the primary outcome, multiple imputation will be undertaken in order to present a sensitivity analysis of the full intention-to-treat population.

The primary analysis of the primary outcome will be undertaken blinded to allocated group, and will involve comparison of the change in overall MANSA score between baseline assessment and 10-month follow-up between allocated groups. The comparison will be undertaken using a mixed-effects linear regression model adjusted for baseline MANSA score, as well as the stratification factors (region and practice size), and including a random effect term for GP practice to account for the hierarchical structure of the data induced by the cluster randomisation. Results without adjustment for the stratification variables will also be presented. Model assumptions will be checked and appropriate data transformations sought if required. Continuous secondary outcomes will be analysed similarly. All treatment effect estimates will be presented alongside 95% CIs. Additional secondary analyses of the primary outcome will be considered to explore the effect of intervention fidelity and Care Partner availability on the estimated treatment effect, using Complier Average Causal Effect and per-protocol analyses.

### Health economics evaluation

The economic evaluation will assess the costs, outcomes and relative cost-effectiveness of the intervention from the perspective of health and social care services (costs) and patients (health benefits), over the 10-month time horizon of the trial. The primary measure of benefit will be the quality adjusted life years, derived from the EQ-5D-5L, and published utility values recommended by NICE at the time of analysis.

NHS and social care costs will be calculated using the participant questionnaire data and data from the audit of primary care records. This includes hospital inpatient and outpatient services, community-based mental health and social care services and primary care services. Service use data will be combined with the most recent published national unit costs to estimate health and social care costs. Regression analysis will be used to extrapolate the 3-month participant reported service use data to the 10-month time horizon. This will be combined with the audit data to estimate a total cost for each participant.

The cost and health benefit data will be analysed by treatment allocated and include data for all participants whether or not they completed planned care. Single imputation will be used for missing baseline measures of cost, utility, and clinical indicators,^17^ but not missing demographic data. An indicator for missing demographic data will be used. The final imputation strategy will depend on the pattern of missingness. If the data approximate Missing at Random or Missing Completely at Random, multiple imputation from available data will be used, as recommended by Faria *et al* *.* ^18^

Regression analysis will estimate net costs and net quality adjusted life years of collaborative care (compared to treatment as usual). Incremental cost-effectiveness ratios will be calculated. Bootstrapping will be used to generate cost-effectiveness acceptability curves, the probability collaborative care is cost-effective, and net benefit statistics.

Sensitivity analysis will explore the impact on the conclusions of alternative health benefit measures, alternative methods of estimating costs, and alternative methods to account for missing data.

### Process evaluation

The realist process evaluation will assess fidelity to the model, and develop an understanding of how the intervention does or does not work. It builds on earlier work^7,8^ and runs in parallel to the trial.

The key objectives are:

1. to assess the fidelity of the intervention during delivery against the PARTNERS2 theory represented by the logic model (Figure 1) and operationalised by the PARTNERS2 manuals;
2. to assess how any changes in the understanding and behaviour of the Care Partners over the duration of the trial were influenced by the initial and top-up training, supervision sessions, and tape-assisted recall;
3. to achieve a more in-depth understanding of how the intervention works or does not work in comparison to the programme theory, and how it can be implemented in different contexts;
4. to develop implementation recommendations, especially in relation to acceptability, adoption, feasibility, fidelity, and penetration; and
5. to understand the impact of delivering the intervention under COVID-19 on the above.

Data will be collected in the following ways:

- semi-structured interviews with service users, carers, practitioners, and PARTNERS2 researchers;
- audio recordings of clinical sessions and follow-up interviews with participant and Care Partner;
- Care Partner reflective practice logs;
- researcher observations and field notes;
- process of care data recording, for example, the nature and frequency of practitioner contact and liason with other bodies;
- survey of fidelity of service received; and
- contextual questionnaires detailing characteristics of practitioners and practices.

In order to encompass the complexity of the PARTNERS2 intervention, the authors are conducting longitudinal, integrated mixed-methods case studies at two levels:

- service user level; and
- Care Partner level.

This realist process evaluation will provide both a refined programme theory and produce evidence that can inform effective interventions for implementation.^19^

### Trial management and independent oversight committees

The Trial Management Group (CI/principal investigator [PI]), trial team, members of Peninsula Clinical Trials Unit (PenCTU)) meet regularly to ensure successful implementation of the trial. The TSC meets annually. The DMC reviewed blinded data and were asked to recommend whether or not, based on the accruing trial data together with any emerging evidence from other relevant research, recruitment should continue. The DMC also considered the detailed blinded sample size review. The TSC and DMC operate in accordance with trial specific charters.

### Ethics

The trial is conducted in accordance with the UK Policy Framework for Health and Social Care Research, the Data Protection Act 2018, and the Principles of Good Clinical Practice. The protocol and subsequent amendments were ratified by the sponsor before being submitted to and approved by the West Midlands-Edgbaston Research Ethics Committee prior to circulation (reference: 14/WM/0052).

#### Consent and confidentiality

Consent was obtained by the programme grant research team and clinical research network-funded staff. Participant information that identifies the participants by name (for example, consent forms, contact forms) will be kept in lockable cabinets or electronic secure data storage. This will be stored separately from trial data, which will be anonymous and confidential. There will be limits to confidentiality where data indicates a risk of harm to the participant or others. At the end of the trial, data will be moved to secure storage for at least 10 years, after which it will be destroyed.

#### Dissemination

Results will be disseminated in peer review journals and local, national, and international conferences. The final report will be submitted to the funder. Plain language summaries will be disseminated to participants in consultation with the LEAPs. Authors will be recognised in the accordance with the predefined PARTNERS2 publication policy. The protocol will be made publically available via the programme webpage.

## Discussion

### Summary

Collaborative care is a system that offers a new way of supporting people with ongoing mental health needs, that may improve primary care engagement and healthcare access impacting on physical health, mental health, and social recovery, for example, returning to vocational and social roles. The vast majority of work on collaborative care has been developed and evaluated in the US, where the nature of service user populations and of service use differ from the way the UK fund, structure, and use the NHS in England. While there is considerable evidence for its effectiveness, this research has largely been carried out with populations with non-psychotic disorders, of mild to moderate severity, and less functional impairment than experienced by people with schizophrenia or bipolar. PARTNERS2 is the first trial of that model to be carried out in the UK.

### Strengths and limitations

- The study is a four-area cluster RCT of a collaborative care coaching intervention for individuals with psychosis; it will provide much-needed evidence of what works for this population, whose needs are often not met.
- The components of the intervention and trial procedures were developed with extensive feasibility work. Primary and secondary outcomes have been selected cover a broad range of outcome domains that could be impacted by the complex intervention.
- The study adopts a flexible and pragmatic approach to recruitment and data collection to try to overcome the challenges of varied NHS services and the COVID-19 pandemic.
- The lack of blinding of researchers collecting study data is a limitation of the study design.

### Implication for research and practice

The aim of the study is to provide an evidence based (social/mental health, effectiveness, and economic) rationale as to how and why the PARTNERS2 intervention should be commissioned.

## Trial registration number

ISRCTN 95702682 (16 October 2017)
